# Emphysematous cholecystitis: a case report

**DOI:** 10.1186/1757-1626-1-73

**Published:** 2008-08-07

**Authors:** Theodossis S Papavramidis, Antonis Michalopoulos, Vassilis N Papadopoulos, Daniel Paramythiotis, Vassiliki Karadimou, Haralambos Kokkinakis, Epameinontas Fahantidis

**Affiliations:** 1 1st Propedeutic Surgical Department, A.H.E.P.A University Hospital, Aristotle's University of Thessaloniki, Thessaloniki, Greece; 2 Radiological Department of AHEPA Hospital, Aristotle's University of Thessaloniki, Thessaloniki, Greece

## Abstract

A 65-year-old Greek man with a history of diabetes mellitus and hypertension was admitted because of right upper quadrant pain, nausea and palpable right quadrant mass. On admission the patient was febrile (38.8°C) with a total bilirubin level of 1.99 mg/dl (direct 0.59 mg/dl); SGOT 1.26 mg/dl; Na 135 mmol/l and K 2.9 mmol/l. The white blood count was 15200/μl with 92.2% neutrophiles. Axial sections of single slice CT imaging (section thickness 10 mm), revealed emphysematous cholecystitis with thickening of gallbladder wall and wall enhancement after iv contrast enhancement, as well as, dilatation of the gallbladder with multiple gallstones precipitate and intraluminal air. The patient underwent subtotal cholecystectomy and a cholecystostomy was placed. The culture of the bile showed positivity to toxin A of Clostridium Difficile and to Escherichia Coli. The postoperative course of the patient was uneventful.

## Background

Emphysematous cholecystitis (E.C) is an uncommon variant of acute cholecystitis in which the causative organisms are gas-forming bacteria. E.C has been defined clinically by the imaging demonstration of air in the gallbladder lumen; in the wall, or in the tissues adjacent to the wall of the gallbladder; and elsewhere in the biliary ducts in the absence of an abnormal communication with the gastrointestinal tract [1]. E.C is pathophysiologically different from acute or chronic cholecystitis. Obstruction of the gallbladder neck secondary to cholelithiasis induces acute and chronic cholecystitis. However, E.C mostly results from thrombosis or occlusion of the cystic artery with ischemic necrosis of the gallbladder wall.

The aim of the present article is to present a case of EC and to attempt to elucidate the clinical entity and management of emphysematous cholecystitis.

## Case presentation

**Figure 1:**
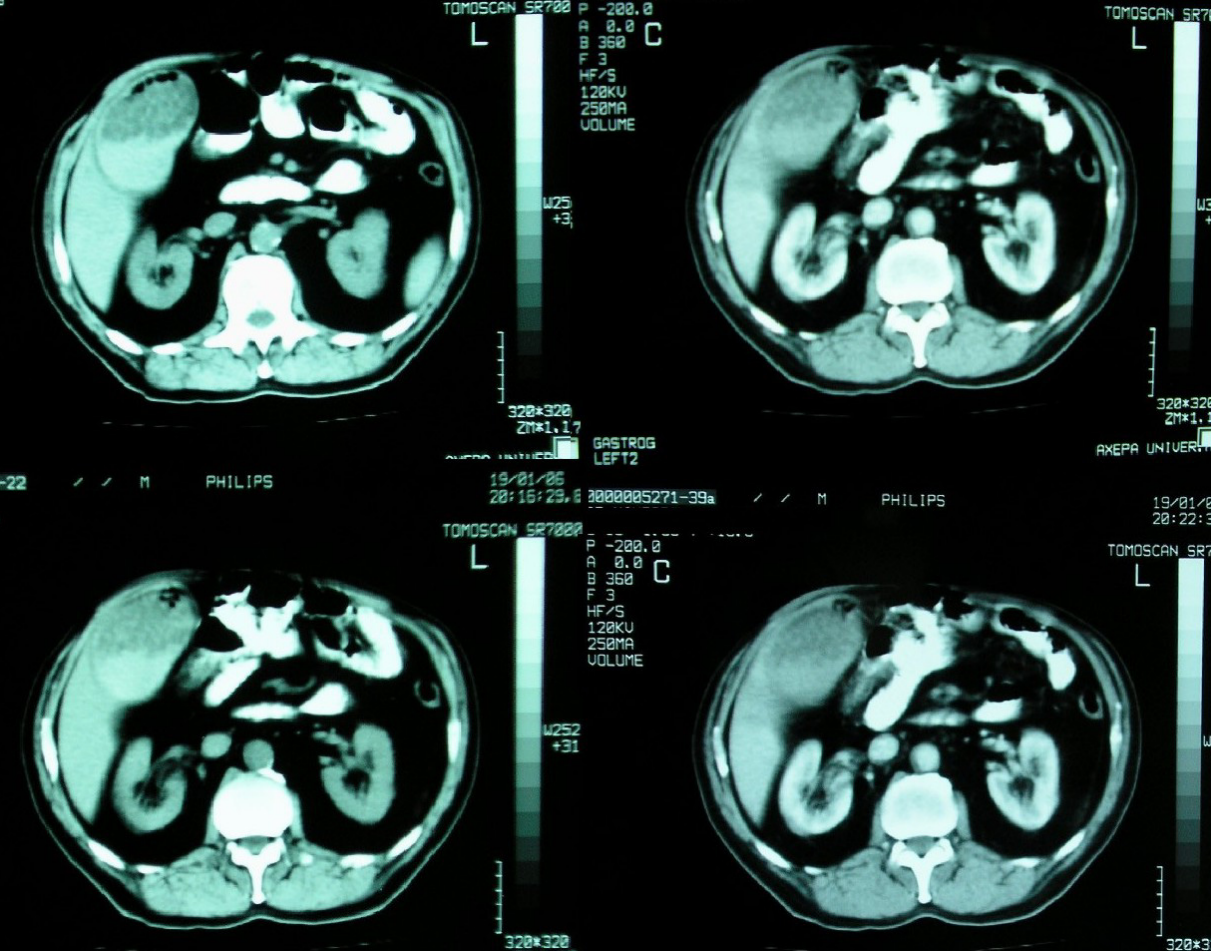
Axial sections of single slice CT imaging (section thickness 10 mm), revealed emphysematous cholecystitis with thickening of gallbladder wall and wall enhancement after iv contrast enhancement, as well as, dilatation of the gallbladder with multiple gallstones precipitate and intraluminal air.

A 65-year-old man with a history of diabetes mellitus and hypertension was admitted because of right upper quadrant pain, nausea and palpable right quadrant mass. On admission the patient was febrile (38.8°C) with a total bilirubin level of 1.99 mg/dl (direct 0.59 mg/dl); SGOT 1.26 mg/dl; Na 135 mmol/l and K 2.9 mmol/l. The white blood count was 15200/μl with 92.2% neutrophiles. Axial sections of single slice CT imaging (section thickness 10 mm), revealed emphysematous cholecystitis with thickening of gallbladder wall and wall enhancement after iv contrast enhancement, as well as, dilatation of the gallbladder with multiple gallstones precipitate and intraluminal air (Figure 1). The patient underwent subtotal cholecystectomy [2] and a cholecystostomy was placed. The culture of the bile showed positivity to toxin A of Clostridium Difficile and to Escherichia Coli. The postoperative course of the patient was uneventful.

## Discussion

Emphysematous cholecystitis (EC) is a virulent form of acute cholecystitis accompanied by gas formation [1]. E.C frequently affects elderly men, and it is associated with diabetes mellitus. The risk of gangrene and perforation of the gallbladder is relatively high for patients with E.C, and the mortality rate is 15% [1].

The presenting symptoms of EC are usually very vague and initially indistinguishable from those of uncomplicated acute cholecystitis, frequently causing a diagnostic dilemma [3]. The clinical presentation may adopt different forms, from minimal pain to septic shock [4]. The succeeding symptoms and signs depend on the evolution of the disease [5].

The diagnosis of E.C is based on the demonstration of varying amounts of gas in the gallbladder lumen and wall, and occasionally in the bile ducts [6]. Intraluminal gas is depicted as one or several round bubbles or a pear-shaped lucency in the right upper quadrant on a supine film or on an air-fluid level within the gallbladder on an erect or decubitus radiograph [7]. Computed tomography is the most sensitive modality for the detection of the intraluminal or intramural gallbladder gas [8], and it can also demonstrate local complications, such as pericholecystic inflammatory changes, abscess formation, or perforation [9]. Prompt diagnosis is essential, as early intervention can minimize the serious morbidity and mortality rates associated with emphysematous cholecystitis.

CT might be the best technique for diagnosing EC because it shows the exact location of air, whether in the gallbladder wall, in the gallbladder lumen, or throughout the bile duct, but it is irrational to perform CT for all patients with vague abdominal symptoms [8]. Ultrasonography (US) is now the first diagnostic tool for observing the gallbladder. Thus, in a clinical setting, US can be used to diagnose EC early and correctly and when the examination is positive it should be followed by CT. US findings of EC are thought to depend on the amount and location of gas.

The treatment proposed by most authors is cholecystectomy, either conventional or laparoscopic. As an alternative technique percutaneous cholecystectomy may be used, if the patient's situation is not good enough for surgical treatment [2]. However, occasional cases of total recovery solely with conservative therapy (intravenous fluids, antibiotics and analgesia) have been reported. All authors agree that a rapid clinical deterioration, especially with the presence of a palpable mass in the right upper quadrant, warrants immediate intervention.

The pathogens responsible for the gas formed in EC are usually anaerobes like Clostridium, or other microorganisms like E. Coli, P. Vulgaris, A. Aerogenes, Staphylococcus, Streptococcus, Klebsiella and B. Fragilis that under special conditions, are able to produce gas [10]. Both Clostridium and E. Coli are common bacteria present in the gastrointestinal tract, especially in the colon and small bowel, or even in the duodenum when local aggressive factors take place (peptic ulcer, digestive surgery e.t.c.). Thereafter, they can migrate from the duodenum to the biliary tract, and it is common to find these microorganisms in bile cultures when there is any pathology in the region [10], although gas production is rare. It may be possible that these bacteria acquire pathogenicity if they proliferate in a previously altered gallbladder. Nevertheless, anaerobic bacteria are the most frequent gas forming microorganisms, and they proliferate only in areas with a poor irrigation, and consequently, with a low oxygen saturation.

## Conclusion

In conclusion, emphysematous cholecystitis is a rare form of cholecystitis that carries a high mortality. Vascular occlusion can be very important in the development of the disease. Ultrasonography has to be performed to all patients, despite the fact that CT is the most accurate imaging technique. Antibiotic therapy should begin quickly and include coverage of common pathogens, particularly Clostridia. Surgical intervention should take place as early as possible with consideration of the patient's potential for deterioration.

## Competing interests

The authors declare that they have no competing interests.

## Authors' contributions

TSP: 1) Received the patient to the emergency department. Advising doctor 2) Involved in drafting the manuscript and revising it critically for important intellectual content. 3) Have given final approval of the version to be published. AM: 1) Main surgeon. 2) Have been involved in revising the draft critically for important intellectual content. 3) Have given final approval of the version to be published. VNP: 1) Auxiliary surgeon. 2) Have been involved in revising the draft critically for important intellectual content. 3) Have given final approval of the version to be published. DP: 1) Auxiliary surgeon. 2) Have been involved in revising the draft critically for important intellectual content. 3) Have given final approval of the version to be published. VK: 1) Preoperative radiologic evaluation, received the patient to the emergency department 2) Have been involved in revising the draft critically for important intellectual content. 3) Have given final approval of the version to be published. HK: 1) Responsible for the radiologic exams 2) Have been involved in drafting the manuscript. 3) Has given final approval of the version to be published. EF: 1) Strategic planning for the treatment of the patient. 2) Has been involved in revising the draft critically for important intellectual content. 3) Has given final approval of the version to be published.

## Consent

Written informed consent was obtained from the patient for publication of this case report and accompanying images. A copy of the written consent is available for review by the Editor-in-Chief of this journal.
